# Osteoporosis in Black South Africans With Rheumatoid Arthritis

**DOI:** 10.7759/cureus.47743

**Published:** 2023-10-26

**Authors:** Tania Naidoo, Lai-Ling Winchow, Mohammed Tikly, Nimmisha Govind

**Affiliations:** 1 Department of Internal Medicine, University of the Witwatersrand, Johannesburg, ZAF; 2 Division of Rheumatology, Chris Hani Baragwanath Academic Hospital, University of the Witwatersrand, Johannesburg, ZAF

**Keywords:** functional class, arthritis, rheumatoid, africans, osteoporosis

## Abstract

Background

Osteoporosis is a common comorbidity associated with rheumatoid arthritis (RA). The aim of this study was to determine the risk factors and possible predictors of osteoporosis in black patients with RA.

Methods

A retrospective study of 120 randomly selected RA patients attending an arthritis clinic in Johannesburg, South Africa, was carried out, in which 60 patients were with and 60 without osteoporosis. The demographics, disease activity, American College of Rheumatology (ACR) functional status, treatment, and dual-energy X-ray absorptiometry (DEXA) characteristics were compared. Statistical analysis was performed using SPSS version 25.0 (IBM Corp, Armonk, NY). Bivariate comparisons of demographic factors, disease factors, and T-scores between patients with and without osteoporosis were performed, using two-sided t-tests for continuous variables and chi-squared tests for categorical variables. Possible predictors of osteoporosis were subsequently entered into a multivariate logistic regression model with osteoporosis being the dependent variable. The level of significance for all analyses was set at p < 0.05.

Results

The median (IQR) age of the overall cohort was 67 (61.0, 72.8) years, the majority (95.5%) were female, of which 97.4% were postmenopausal. The mean disease duration from diagnosis to the DEXA was 8.6 ± 6.2 years. Rheumatoid factor (RF) positivity was 89.2% and anti-cyclic citrullinated peptide (ACCP) positivity was 82.7%. The median (IQR) for disease activity score 28 swollen and tender joint count using the erythrocyte sedimentation rate (DAS-28 ESR) was 3.4 (2.8-4.7) and the median (IQR) for ESR was 41 (22, 64.3) mm/h. There were significantly more patients treated with triple therapy in the no osteoporosis group, 38 (63.3%), than in the osteoporosis group, 21 (35%) (p = 0.00). The ACR functional class was significantly worse in the RA patients with osteoporosis than in the RA patients without osteoporosis [median (IQR), 2 (2, 3) vs 2 (1, 2), (p = 0.03)], respectively.

Conclusion

This study found that a worse ACR functional class was significantly associated with osteoporosis. In addition, the use of triple therapy had a protective effect. Early recognition of the risk factors for osteoporosis should be sought, with prompt preventative measures, screening, and treatment.

## Introduction

Rheumatoid arthritis (RA) is a prevalent inflammatory joint disease affecting around 1% of adults globally [1]. It is a destructive joint disease resulting in substantial pain, significant disability, and impairment of quality of life if untreated. Osteoporosis develops when there is reduced bone mass which results in an increased risk of fractures which can lead to substantial pain, disability, and an increased economic burden. The association between RA and osteoporosis is well documented, with osteoporosis being more prevalent in RA patients compared to the general population [2].

In RA patients, women have a twofold increase in osteoporosis while men show a twofold risk of reduced bone mass compared to patients without RA [3]. The prevalence of osteoporosis varies across different RA populations, ranging from 22% in India [4] to 46.8% in Korea [2]. The Italian Study Group found a frequency of osteoporosis in patients with RA of 28.8% at the lumbar spine and 36.2% at the femoral neck [5]. The Total Management of Risk Factors in RA patients to Lower Morbidity and Mortality (TOMORROW) study found the prevalence of vertebral fractures to be as high as 45.5% in patients with RA [6].

Osteoporosis development in RA is influenced by both traditional risk factors such as age, gender, and low body mass index and RA-specific risk factors. Factors such as high disease activity, prolonged disease duration, and poor functionality as measured by the Health Assessment Questionnaire (HAQ) contribute to osteoporosis risk in RA [2,7]. Proinflammatory cytokines such as TNF-α, IL-1, IL-6, and IL-17 that are upregulated in RA enhance osteoclastogenesis [8,9]. Rheumatoid factor (RF) and anti-citrullinated cyclic peptide (ACCP) antibodies are not only pathogenic in RA development but also contribute to osteoporosis [9]. Glucocorticoids, commonly used to manage inflammation, are associated with increased fracture risk in RA patients with the fracture risk in RA patients with early disease exposed to glucocorticoids as compared to non-exposed RA patients being doubled [10].

The development of fractures in RA leads to a decline in the quality of life and a substantial increase in the costs of rehabilitation and treatment. Osteoporotic fractures yield an enormous economic burden, from the direct burden of hospitalizations and physician visits, which in 2002 ranged from 12.2 to 17.9 billion dollars per year, as well as indirect costs, related to decreased productivity due to morbidity and premature mortality [11]. The mortality rate from fractures as a result of osteoporosis is higher than mortality from other causes such as cervical cancer, uterine cancer, and breast cancer [12].

This study aimed to identify the risk factors for osteoporosis in black South African RA patients attending a tertiary care facility. The study analyzed patient demographics, traditional osteoporosis risk factors, disease activity, autoantibodies, functional status, and disease-modifying antirheumatic drug (DMARD) therapy. This study was approved by the Human Research Ethics Committee of the University of the Witwatersrand (M170705).

## Materials and methods

A retrospective study of 120 RA patients attending an arthritis clinic in Johannesburg, South Africa, was performed. All patients were adults (≥18 years of age at disease diagnosis), fulfilled the 1987 American College of Rheumatology (ACR) criteria for RA [13], and had dual-energy X-ray absorptiometry (DEXA) scans done. Of the 120 randomly selected RA patients studied, 60 had osteoporosis and 60 had no osteoporosis.

Data collected were patient demographics, traditional osteoporosis risk factors such as smoking and significant alcohol use defined as ≥3 units per day, history of fractures, symptom duration prior to DEXA, RA disease activity, autoantibodies, ACR functional status, and DMARD therapy. Autoantibodies included RF and ACCP. Disease activity was determined at the visit prior to the DEXA using DAS-28 ESR [14]. The physical function was evaluated using the 1991 ACR functional classification (FC) [15]. Disease activity markers ESR, CRP, and the ACR FC were taken in the visit closest to the DEXA scan but not more than a year before.

Osteoporosis was defined as a T-score ≤2.5 SD at any of the three sites (total hip, femoral neck, or spine) measured using the WHO criteria [16]. The DMARD escalation strategy starts with monotherapy methotrexate (MTX), then with combination therapy with MTX, sulphasalazine, and chloroquine (CHQ) (triple therapy) if not in remission or low disease activity defined by DAS-28 ESR. Leflunomide is used if combination therapy fails.

Statistical analysis was performed using SPSS version 25.0 (IBM Corp, Armonk, NY). Bivariate comparisons of demographic factors, disease factors, and T-scores between patients with and without osteoporosis were performed using two-sided t-tests for continuous variables and chi-squared tests for categorical variables. Possible predictors of osteoporosis were subsequently entered into a multivariate logistic regression model with osteoporosis being the dependent variable. The level of significance for all analyses was set at p < 0.05.

## Results

The median (IQR) age of the overall cohort was 67 (61.0, 72.8) years with the majority being postmenopausal females, 111/120 (97.4). The disease duration from diagnosis to the DEXA scan was eight years. A minority consumed alcohol, 7 (6.1%), or were ever smokers, 11 (9.6%). A previous osteoporotic fracture was recorded in only 8 (7%) patients. Most of the patients were seropositive; RF positivity was 89.2% and ACCP positivity was 82.7% and had moderate disease activity with a DAS-28 ESR, median (IQR) of 3.4 (2.8, 4.7) prior to the DEXA scan (Table 1). There was a total of eight previous fractures in the entire cohort; however, it is not known if these were fragility fractures.

**Table 1:** Demographics, clinical features, and drug therapy in 120 rheumatoid arthritis patients with and without osteoporosis OP = osteoporosis, SD = standard deviation, IQR = interquartile range, RA = rheumatoid arthritis, RF = rheumatoid factor, ACR = American College of Rheumatology, DAS-28 ESR = disease activity score 28 swollen and tender joint count using the erythrocyte sedimentation rate, ACPA = anti-citrullinated protein antibody.

Variable	All patients (n=120)	RA with OP (n-60)	RA without OP (n=60)	OR (95% CI)	p-Value
Female gender, n (%)	114 (95.0)	59 (98.3)	55 (91.7)	5.4 (0.6, 47.4)	0.20
Age in years, median (IQR)	67.0 (61.0, 72.8)	68.0 (63.0, 73.8)	66.0 (57.2, 71.0)	-	0.088
Disease duration from diagnosis to DEXA in years, median (IQR)	8 (4, 13)	8 (4, 14)	8 (4.2, 12)	-	0.62
Postmenopausal, n (%)	111 (97.4)	57 (96.6)	54 (98.2)	0.53 (0.05, 5.99)	1.00
Alcohol history (>3 units per day), n (%)	7 (6.1)	5 (8.6)	2 (3.5)	2.59 (0.48, 13.96)	0.44
Smoking history, n (%)	11 (9.6)	6 (10.3)	5 (8.8)	1.2 (0.34, 4.18)	1.00
RF positive, n (%)	107 (89.2)	56 (93.3)	51 (85.0)	2.47 (0.72, 8.52)	0.24
Anti-ACPA, n (%)	81 (82.7)	46 (82.1)	35 (83.3)	0.92 (0.32, 2.66)	1.00
DAS28-ESR, median (IQR)	3.4 (2.8, 4.7)	3.3 (2.8, 4.8)	3.7 (2.7, 4.7)	-	0.95
ESR mm/h, median (IQR)	41 (22, 64.3)	41.5 (22.5, 70.8)	41 (21.3, 60.8)	-	0.67
ACR functional class, median (IQR)	2 (1, 2)	2 (2, 3)	2 (1, 2)	-	0.03
Previous history of fractures	8 (7.0)	5 (8.6)	3 (5.3)	1.7 (0.39, 7.46)	0.72
HIV positive, n (%)	4 (3.4)	3 (5.0)	1 (1.7)	3.05 (0.31, 30.22)	0.62
Drug therapy					
Prednisone, n (%)	100 (83.3)	50 (83.3)	50 (83.3)	1 (0.38, 2.61)	1.00
Prednisone >7.5 mg, n (%)	24 (24.0)	9 (18.0)	15 (30.0)	0.51 (0.2, 1.31)	0.24
Prednisone use (duration in months), median (IQR)	25.5 (12, 57)	28 (10, 61.3)	25 (16, 51.5)	-	0.68
Methotrexate, n (%)	112 (93.3)	56 (93.3)	56 (93.3)	1 (0.24, 4.2)	1.00
Chloroquine, n (%)	83 (69.2)	37 (61.7)	46 (76.7)	0.49 (0.22, 1.08)	0.11
Sulphasalazine, n (%)	75 (62.5)	32 (53.3)	43 (71.7)	0.45 (0.21, 0.96)	0.06
Triple Rx	59 (49.2)	21 (35)	38 (63.3)	0.31 (0.15, 0.66)	0.00
Leflunomide, n (%)	43 (35.8)	20 (33.3)	23 (38.3)	0.8 (0.38, 1.7)	0.70

Most patients (83.3%) were treated with corticosteroids for an average of two years. The majority were treated with MTX, approximately half were on triple therapy, and a third of patients were on leflunomide. There were significantly more patients treated with triple therapy in the no osteoporosis group (63.3%) than in the osteoporosis group (35%) (p = 0.00).

The ACR FC was significantly worse in the RA with osteoporosis group than in the RA without osteoporosis group [median (IQR), 2 (2, 3) vs 2 (1, 2), (p=0.03)], respectively.

In the osteoporosis group, T-scores were worse at the spine than at the femoral neck and total hip. Osteopenia was frequently recorded in the non-osteoporosis group (Table 2).

**Table 2:** DEXA characteristics of 120 rheumatoid patients with and without osteoporosis RA = rheumatoid arthritis, OP = osteoporosis, DEXA = dual-energy X-ray absorptiometry.

Variable	All patients (n = 120)	RA with OP (n = 60)	RA without OP (n = 60)	OR (95% CI)	p-Value
Femoral neck					
T-score, median (IQR)	-1.7 (-2.33, -0.68)	-2.30 (-2.80, -1.80)	-0.80 (-1.58, 0.45)	-	<0.0001
Osteopenia, n (%)	54 (45.8)	29 (50.0)	25 (41.7)	1.4 (0.68, 2.9)	0.46
Osteoporosis, n (%)	26 (22)	26 (44.8)	0 (0.0)	-	0.000
Total hip					
T-score, median (IQR)	-1.5 (2.30, -0.30)	-2.25 (-2.83, -1.60)	-0.5 (-1.28, 0.40)	-	<0.0001
Osteopenia, n (%)	46 (39)	27 (46.6)	19 (31.7)	1.88 (0.89, 3.98)	0.131
Osteoporosis, n (%)	25 (21.2)	25 (43.1)	0 (0.0)		0.000
Spine					
T-score, median (IQR)	-2.1 (-3.00, -0.90)	-3.00 (-3.60, -2.60)	-1.10 (-1.70, 0.10)	-	<0.0001
Osteopenia, n (%)	40 (33.6)	9 (15.0)	31 (52.5)	0.16 (0.07, 0.38)	<0.0001
Osteoporosis, n (%)	47 (39.5)	47 (78.3)	0 (0.0)	-	0.000

Multivariate logistic regression showed that combination therapy with MTX, CHQ, and sulphasalazine was significantly protective of osteoporosis, OR = 3.9 (1.1-8.6), p = 0.001. Poor functional class was also a significant predictor of osteoporosis (OR = 1.9 (1.1-3.6), p = 0.022) (Table 3).

**Table 3:** Predictors of osteoporosis ACR = American College of Rheumatology.

Predictors of osteoporosis	OR (95% CI)	p-Value
Triple therapy	3.87 (1.75, 8.55)	0.001
ACR functional class	1.99 (1.10, 3.58)	0.022

**Figure 1:**
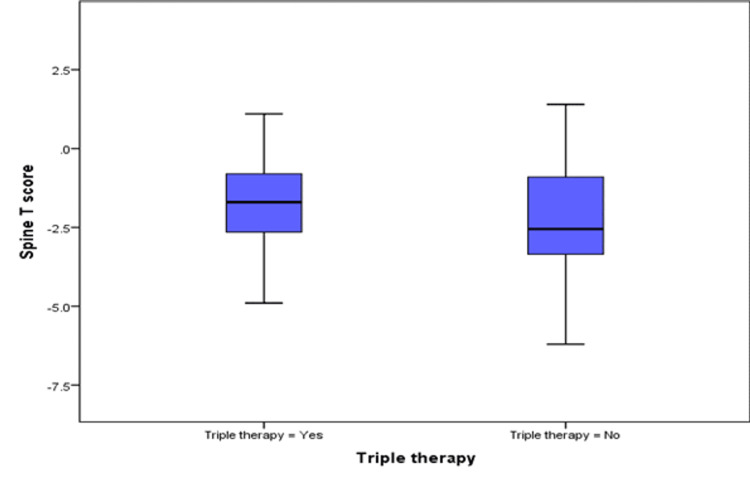
Spine T-scores of patients treated with and without triple therapy.

**Figure 2:**
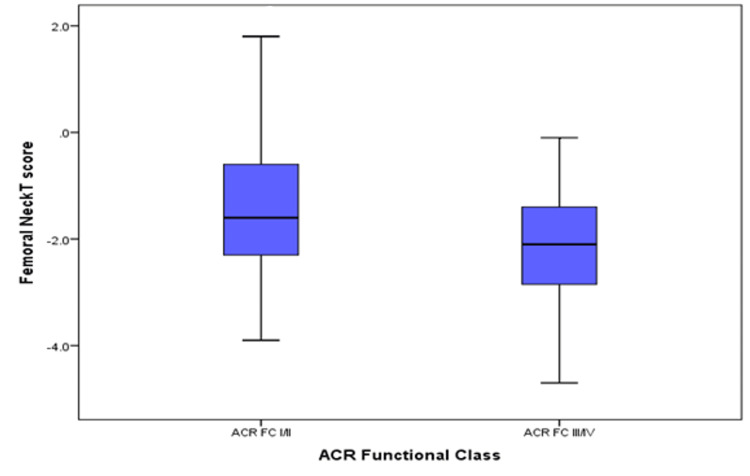
Femoral neck T-scores of patients in different functional classes ACR = American College of Rheumatology.

Higher spine T-scores were observed in patients on triple therapy as was seen in patients with better functionality (ACR functional class I, II). The median (IQR) spine T-score for patients on triple therapy was statistically higher, -1.70 (-2.70, -0.80), compared to those not on triple therapy, -2.55 (-3.38, 0.90), p-value 0.023 (Figure 1). The median (IQR) femoral neck T-scores for patients with ACR FC I/II were statistically higher, -1.60 (-2.30, -0.80), compared to patients with ACR FC III/IV -2.10 (-2.93, -1.40), p-value 0.041 (Figure 2).

## Discussion

Similar to other studies, osteoporosis and osteopenia were common in this cohort of black South Africans with RA. We identified poor functionality as a predictor of osteoporosis and triple DMARD therapy as protective. To the best of our knowledge, there are very few studies describing risk factors for osteoporosis in patients with RA in South Africa. 

Many studies have demonstrated that RA patients have a substantially increased fracture risk than patients with no RA. These studies vary with regard to ethnicity (Caucasian and Asian), gender, and the number of patients recruited with sample sizes varying from 14 to 47,034 RA patients [12]. Chen et al. [17] conducted a meta-analysis of studies of RA patients from the UK, Japan, Sweden, and France of both genders and ages ranging from 40 to 79 years, which showed the relative risk (pooled) of fracture at the vertebrae in RA patients to be 2.34.

Like other studies [2,18] our study showed ESR and DAS-28 ESR not to be independent risk factors for osteoporosis. This is in contrast to other studies [19,20] that showed that higher ESR was statistically associated with the occurrence of osteoporosis.

Similar to this study, poor functionality in RA has been associated with osteoporosis in other studies [2,5,20]. Worse modified health assessment questionnaire (mHAQ) scores were found to be an independent risk factor for osteoporosis [21] with bone loss almost doubling in the lumbar spine and in the femoral neck in patients with high HAQ scores. Despite using a different measure of physical function, the ACR FC, we found poorer functionality to be associated with osteoporosis. 

Glucocorticosteroids are an important cause of osteoporosis in RA with 10% of patients on long-term treatment developing fractures and radiographic evidence of vertebral fractures being found in 30-40% of patients [22]. Despite the average duration of steroid use being over two years, we found no association with osteoporosis in our study. The study was limited as we were unable to calculate the cumulative dose.

The use of triple therapy, i.e. MTX, sulphasalazine, and CHQ, was significantly protective against the development of osteoporosis. This may be explained by better disease activity control although this was not reflected by the ESR or the DAS-28 ESR. Although a study of children whose mothers were treated with sulphasalazine had higher total body bone mineral density (BMD) [23], other studies have not shown any association between MTX either alone or in combination with other DMARDs and incident fractures or BMD [24]. We postulate that this may be because disease activity measures were taken at one point in time rather than an average value over time.

There are several limitations to the study. The retrospective cross-sectional nature of this study is a major shortcoming with respect to missing data and inconsistencies in documenting clinical problems in case records. Furthermore, a majority of the patients were postmenopausal women and therefore cannot be generalized to all RA patients. Also, we were unable to quantify the cumulative prednisone dose or assess vertebral fractures.

## Conclusions

Osteoporosis and therefore fragility fractures are more frequent in RA. This study found that worse ACR functional class was significantly associated with osteoporosis in RA patients. In addition, the use of triple therapy had a protective effect. Early recognition of the risk factors for osteoporosis should be sought, with prompt preventative measures, screening, and treatment.
